# The Association Between Intestinal Bacteria and Allergic Diseases—Cause or Consequence?

**DOI:** 10.3389/fcimb.2021.650893

**Published:** 2021-04-15

**Authors:** Pei Han, Jian-Qing Gu, Li-Sha Li, Xue-Yan Wang, Hong-Tian Wang, Yan Wang, Christopher Chang, Jin-Lyu Sun

**Affiliations:** ^1^ Allergy Department, State Key Laboratory of Complex Severe and Rare Diseases, Peking Union Medical College Hospital, Chinese Academy of Medical Sciences and Peking Union Medical College, Beijing, China; ^2^ State Key Laboratory of Bioactive Substance and Function of Natural Medicines, Institute of Materia Medica, Chinese Academy of Medical Sciences/Peking Union Medical College, Beijing, China; ^3^ Department of Allergy, Beijing Shijitan Hospital, Capital Medical University, Beijing, China; ^4^ Division of Rheumatology, Allergy and Clinical Immunology, University of California, Davis, Davis, CA, United States; ^5^ Division of Pediatric Immunology and Allergy, Joe DiMaggio Children’s Hospital, Hollywood, FL, United States

**Keywords:** allergic disorders, gut microbiome, advanced techniques, therapeutic interventions, interactions

## Abstract

The incidence of allergic disorders has been increasing over the past few decades, especially in industrialized countries. Allergies can affect people of any age. The pathogenesis of allergic diseases is complex and involves genetic, epigenetic, and environmental factors, and the response to medication is very variable. For some patients, avoidance is the sole effective therapy, and only when the triggers are identifiable. In recent years, the intestinal microbiota has emerged as a significant contributor to the development of allergic diseases. However, the precise mechanisms related to the effects of the microbiome on the pathogenesis of allergic diseases are unknown. This review summarizes the recent association between allergic disorders and intestinal bacterial dysbiosis, describes the function of gut microbes in allergic disease development from both preclinical and clinical studies, discusses the factors that influence gut microbial diversity and advanced techniques used in microbial analysis. Ultimately, more studies are required to define the host-microbial relationship relevant to allergic disorders and amenable to new therapeutic interventions.

## Introduction

Allergic disorders have been on the increase in recent years, and especially in industrialized countries. With an epidemic growing, it has now been regarded as one of the “three major diseases of the 21st century” by the World Health Organization (WHO) and “a public health issue of global concern” by World Allergy Organization (WAO) (Cezmi A. Akdis, 2014; Shen et al., 2019). It is estimated that the prevalence of allergic disorders would reach 4 billion worldwide in 2050 and unfortunately we are nowhere near a cure (Cezmi A. Akdis, 2014; American Academy of Allergy Asthma and Immunology, 2018).

The 200^th^ anniversary of the introduction of the concept of allergy, first introduced by John Bostock, a physician at London Guy’s Hospital, in 1819 as “hay fever” occurred last year. It is perhaps quite noteworthy that the mechanism of the pathogenic factors and pathways related to the development and persistence of allergy is still not completely elucidated. The journey has been slow, punctuated by the discovery of grass pollen as a causative agent of allergy in 1873 by Charles Blackley, and the discovery of IgE in 1967 by Johansen and the Ishizakas as the tissue-sensitizing factor related to acute allergic reactions (Holgate, 1999; Galli and Tsai, 2012).

Why the immune system of some individuals reacts dysfunctionally to generally harmless substances such as common environmental allergens while others show no effect is unknown. It is well known that genetics plays a significant role in allergies, but then environment contributes significantly as well (McConnell, 2013; Kubo et al., 2017; D’Auria et al., 2019; Stephen-Victor and Chatila, 2019). Common allergens include pollens, dust mites, insect stings, and certain food, contributing to respiratory, skin, and food allergy (Robinson et al., 2004; British Society for immunology, 2017).

Epidemiological studies revealed that allergic diseases threaten the health of approximately 80% of families and up to 30% of the population globally, reaching epidemic proportions with no signs of abating (Sánchez-Borges et al., 2018; Fyhrquist, 2019). Intriguingly, allergic diseases, such as hay fever, used to be regarded as an aristocratic disorder, as they were often diagnosed among the upper classes of the society or rich educated people, whereas very rarely it occurred to working class population in rural areas. However, the industrialization associated with modern society has played a role in changing this pattern, and with the rise of the middle class, improved living conditions and hygiene, the prevalence has become more evenly distributed (Matricardi et al., 2002; Thomsen, 2015). A similar trend was observed in atopic dermatitis. Nowadays, IgE mediated sensitization to foreign antigens outside the primary indigenous environment occurs in about 40% of the world population (American Academy of Allergy Asthma & Immunology, 2019). In the United Kingdom (UK), the number of allergy cases increases by approximately 5% annually, especially in children (British Society for immunology, 2017). In China, the incidence of allergic rhinitis has increased to 6.5% and continues to increase (Zhang and Zhang, 2019). In the US, more than 50 million people suffer from various allergies every year (American Academy of Allergy Asthma and Immunology, 2018), and it has become the sixth leading cause of chronic illnesses in the United States.

According to the WAO White Book on Allergy, 300 million people in the world have experienced asthma, and about 200 to 250 million people developed food allergies. In addition, one tenth of the total population are affected by drug allergies which is also the main cause for allergy related death (Pawankar, 2014; American Academy of Allergy Asthma and Immunology, 2018). Moreover, the quality of life was reported to be considerably impaired in patients with allergic diseases with reduced functional capacity, leading to diminished work/school performance (Leynaert et al., 2000; Maurer and Zuberbier, 2007). Due to the immense number of affected people together with the resulting direct and indirect costs, allergic diseases have become a major health problem and economic burden. A better understanding of the disease mechanisms involved in allergic diseases is critical to develop prevention and treatment strategies (Cezmi A. Akdis, 2014). Genetic factor is of vital importance in the development of allergic diseases, however they alone are unable to explain the rapid increase over the past century (Abrahamsson et al., 2015).

Over the past 15 years, one of the most remarkable achievements in medical research is our understanding that the gastrointestinal (GI) bacteria profoundly impact human’s health and disease (Ballet et al., 2018; Cani, 2018; Durazzo et al., 2019). Nearly 150 years ago, Robert Koch, the father of modern bacteriology, believed that a person who was not ill could not have bacteria in their bodies. We know now that this doctrine is incorrect. The mucosal surfaces of human body harbor numerous numbers and species of bacteria and other microorganisms. With a bacterial load of more than 10^14^ bacteria, the gastrointestinal tract forms the most complex and diverse ecosystem (Kamada et al., 2013; Thursby and Juge, 2017; GUT MICROBIOTA FOR HEALTH, 2018). The genome carried by gut microbiota outnumbers human genome by 100 times, endowing us with genetic and metabolic functional features that we do not possess on our own (Backhed et al., 2005; Gomes et al., 2014; Thursby and Juge, 2017). This microbial community has a high specificity to the host, coevolving with the host from birth. Both external and internal modifications could elicit structural and functional shifts in the microbial community (Sekirov et al., 2010; Li et al., 2019). In a recent report published in 2019, 1,952 new species of bacteria were discovered, expanding the known species by an increase of 281% phylogenetic diversity (Almeida et al., 2019). The commensal bacteria are highly diverse at species level. The identified bacterial species could be classified into 12 phya, around 90% belonging to Bacteroidetes and Firmicutes (Gomes et al., 2014; Thursby and Juge, 2017).

As far back as 400 BC, Hippocrates stated that “death sits in the bowels” and that “bad digestion is the root of all evil.” In the 1900s, Elie Metchnikoff, a Russian-born zoologist and microbiologist, postulated that gut florae contributed to our health and longevity. Both of the doctrines convey the importance of the intestinal tract in human health (Sekirov et al., 2010; Podolsky, 2012). In modern times, the advent of-omics techniques opened new doors to investigate the impact of the intestinal microbiota in various human disorders (Ballet et al., 2018; Tilg et al., 2019). One study estimates that over 1.7 billion US dollars has been spent on GI microbiota in the past decade (Proctor, 2019). Moreover, the accumulated data suggest the significance of intestinal bacteria in shaping the basic physiology of the human host (Ainsworth, 2020; Eisenstein, 2020).

The microbiome exerts its influence on the biological functions of hosts *via* two ways, directly, and indirectly by producing both useful and harmful compounds from dietary materials (Carding et al., 2015; Hirata and Kunisawa, 2017). Based on their role to human health, this microbial community can be grouped into three categories, namely healthy bacteria (probiotics), unhealthy bacteria (pathogenic bacteria), and health neutral bacteria (neutral bacteria) (Rastall, 2004). Probiotics are “live microorganisms when administered in adequate amounts confer a health benefit on the host” according to WHO whereas pathogenic bacteria can cause damage to the host (Sánchez et al., 2017). Neutral bacteria can switch from probiotics to pathogenic bacteria depending on the surrounding environment or the host health status. Due to the direct contact between commensal microbes and the mucosal surface, there is growing recognition that these microorganisms have profoundly impacted host immune system. They can regulate and stimulate host immune development, and co-existed with it in a remarkable balance (Caruso et al., 2021). Studies have shown that gut microbiota is involved in the immunity development of the organs. It is able to determinate the tendencies of host immune response, regulate type 2 immunity, modulate basophil hematopoiesis, and maintain proper intestinal barrier function (Montecchiani and Fanos, 2020). Moreover, some bacteria-derived metabolites contribute to the immune maturation and development. For example, short-chain fatty acids have an impact on immune homeostasis *via* the modulation of local colonic FOXP3+ regulatory T cells (Caruso et al., 2021) The crosstalk between mucosal immune cells and resident microbiome turn out to be a pivotal element of such an immune balance. This balance is crucial for an optimum immune response without causing an overreaction (Hegazy et al., 2017; Pandiyan et al., 2019). Therefore, dysbiosis has been implicated in the pathogenesis of immune-mediated disorders, such as allergic diseases.

This review aims first to provide the research of the current understanding of gut microbiota and allergic diseases, including advances and updates linking the gut microbiota with allergic diseases, and summarize the factors that lead to gut microbial alterations. Finally, this review will highlight the advanced techniques available to study these relationships.

## Methods

Studies for this review were identified by following the criteria described below: Papers were included by searching electronic databases, i.e., google scholar and PubMed. MeSH terms used in this search were “intestinal microbiota,” “allergic disorders,” and “epidemiology.” Other searches in google scholar or PubMed included the following combinations, in which the MeSH terms of “intestinal microbiota” or “gut microbiota” and (a) “food allergy”; (b) “respiratory allergy,” “allergic rhinitis,” or “asthma”; (c) “skin allergy” or “atopic dermatitis”; (d) “drug allergy” were used to retrieve the number of papers which are studying the allergic disorders and gut microbiota. Another “intestinal microbiota” or “gut microbiota” and (a) “factors”; (b) “genetic factors” or “genotype”; (c) “environment,” “environmental factors,” or “living style”; (d) “delivery mode” or “birth mode”; (e) “breastfeeding”; (f) “nutrition”; (h) “antibiotics” were applied to identify the number of publications discussing the factors which affect gut microbiota. The last search was “intestinal microbiota” or “gut microbiota” and (a) “techniques”; (b) “culture techniques”; (c) “metabolomics,” “transcriptomics,” or “proteomics”; (d) “gene editing”; (e) “mass spectrometry” to identify the number of papers which were published concerning the techniques used to study microbes. Studies involving both human participants and animal models were included. Only studies published in English language were selected. Also a restriction on year of publication was applied to the last 15 years (2005–2020). However, if the reference was cited to explain a concept/technique or a phenomenon, then the restriction on year was not applied.

## Allergic Diseases and Gut Microbiota

In 2017, biologist Erik Wambre and immunologist William Kwok were the first to discover that T helper type 2 cells are specific cells that trigger allergic reactions, and this result was confirmed by a Japanese research team in February 2018 by demonstrating a link between T helper type 2 cells and allergen sensitization in allergic rhinitis (Wambre et al., 2017; Iinuma et al., 2018). Although research on the use of specific immune molecules to prevent allergic diseases has been carried out for more than two decades, no effective strategies have been established (Wahn, 2013). The application of genome-wide association analysis (GWAS) has led to the discovery of dozens of genes involved in the occurrence of allergic diseases (Ferreira et al., 2019; Johansson et al., 2019). Among the genes, mutations in the filaggrin gene (*FLG*) is the most profound single gene isolated that greatly increases the risk of contact allergy, allergic rhinitis, and peanut allergy (Irvine et al., 2011). However, genetic abnormalities do not necessarily result in phenotype differentiation, thus reducing the ability to accurately predict diseases. Other studies have shown that a defect of respiratory epithelial tissue will increase the probability of allergic diseases *via* promoting the penetration of allergens, which in turn leads to Th2-mediated inflammatory reactions (Georas and Rezaee, 2014; Bernard et al., 2015). However, there is still no comprehensive theory that can fully explain the underlying mechanism of the onset and progression of allergic diseases.

In the past decades, multiple studies have described alterations in gut microbiota in allergic individuals, especially in regard to microbial diversity and the relative abundance of specific bacterial strains. In this part, we synthesized the research that indicated a putative causative role for the gut microbiota in allergic diseases progression from both animal models and clinical studies.

## Food Allergy and Gut Microbiota

Food allergy is a pathological immune reaction triggered by certain food proteins. It manifests in a range of disorders within GI tract or/and skins, which could give rise to the most dramatic or even fatal anaphylactic reactions (Du Toit et al., 2018; Renz et al., 2018; Savage et al., 2018). Extensive data has suggested that the incidence of food allergies is increasing at an alarming rate in the last 3 decades and it occurred more often in children than adults (Stefka et al., 2014; Sicherer and Sampson, 2018). Although food allergies are heterogeneous, a short list of foods could encompass the majority of all allergenic foods, namely peanut, wheat, soy, nuts, fish, cow milk, shellfish, eggs, and seeds (Chafen et al., 2010; Sicherer and Sampson, 2018). Among them, peanut is the most common cause of allergy attack and death (Du Toit et al., 2015). In young kids and babies, cow milk and eggs are the most frequently reported food allergens, with egg allergy prevailing in Asia and Australia, and milk allergy in the US and Middle East (Koplin et al., 2015; Tang et al., 2019). Allergies to eggs, soy beans, wheat, and cow milk are often outgrown, whereas peanuts, seeds, seafood, and tree nuts allergies tend to persist into adulthood (Savage et al., 2016). Food allergy is common and pricy. It is estimated that food allergy has affected up to 10% of the population in the developed world and 6–8% of children under 5 years old, which results in costs nearly $4,184 per child on average annually (Savage and Johns, 2015; Willits et al., 2018). The mainstream for the treatment of food allergy is to avoid food allergens strictly whilst the benefit of immunotherapy for food allergy is still ambiguous, hence the procedure is not recommended yet (Kyburz et al., 2017).

The real cause of food allergy is still unclear, but it is widely accepted that alterations in the gut bacterial levels or diversity is the leading reason for the rise of food allergy incidence (Savage et al., 2018). Several landmark studies from the past few years have reported a microbiome signature in food allergy, especially in children, suggesting that dysbiosis in early life may be predictive of disease persistence. The early colonization of gut microbiota in babies has been demonstrated to influence the risk of food allergy at later stages of life. The composition of intestinal microbiome of 3–6 months’ infants was proven to affect milk allergy resolution at the age of 8 years old. The children whose milk allergy resolved later presented a distinct intestinal bacterial composition with enrichment of *Clostridia* and *Firmicutes* after birth compared to those whose allergy to milk persisted (Bunyavanich et al., 2016). Low gut microbiota richness in early infancy and higher *Enterobacteriaceae* to *Bacteroidaceae* ratio (at 3 months) was also shown to be associated with food sensitization at 1 year. Each quartile increase in richness correlated with a 55% reduction in risk for food sensitization by 1 year (Azad et al., 2015). However, currently, there is no specific bacterial profile associated with food allergy. It is possibly because of (a) heterogeneity in the design of study, (b) diverse techniques applied to gut microbiota analysis, and (c) different food allergen leading to distinct bacteria shift (Zhao et al., 2019; Lee et al., 2020). Intestinal dysbiosis in egg-allergic children was characterized by an increase in *Streptococcaceae*, *Lachnospiraceae* genera, and a decrease in *Leuconostocaceae* families compared to non–food-allergic controls. Additionally, metagenome functional analyses of operational taxonomic unit (OTUs) related with egg allergy revealed a decreased purine metabolism in egg allergic subjects. However, no causal relationship was concluded in this study and one limitation of this study was that the control subjects were not completely non-allergic, but with atopic dermatitis (Fazlollahi et al., 2018). As for milk-allergy, in addition to some overlaps, *Lachnospiraceae* and *Ruminococcaceae* were the dominating bacterial strains in the gut microbial community of milk-allergic children (Berni Canani et al., 2016). In a cohort study of 225 children, fecal samples were collected at the age of 3–6 months. The genera of *Dialister*, *Haemophilus*, *Clostridium*, and *Dorea* were found to be reduced in the subjects with food sensitization to at least one food (milk, egg, peanut, soy, wheat, walnut) at age 3. Meanwhile, the genera of *Citrobacter*, *Oscillospira*, *Lactococcus*, and *Dorea* presented strong association with food allergy, all of which were under-expressed in children who had food allergy (Savage et al., 2018).

Intriguing discoveries made from animal model also imply the role of intestinal microbes in food allergy. One study demonstrated that egg ovalbumin sensitized mice displayed a distinct gut abundance of *Lachnospiraceae*, *Lactobacillaceae*, *Rikenellaceae*, and *Porphyromonadaceae* compared to the wild-type mice. When the germ-free wild-type (GFWT) mice were reconstituted with the microbiota from the egg ovalbumin-sensitized mice, the disease susceptibility was also transited. The GFWT mice suffered anaphylaxis upon egg ovalbumin challenge as evidenced by higher OVA-specific IgE response and related symptoms (Noval Rivas et al., 2013). A recent study also observed a similar phenomenon. Feehley and his colleagues transferred feces from healthy infants or infants who are allergy to cow’s milk to germ-free mice. The mice received feces from healthy infants were protected against sensitization to the cow’s milk allergen. Additionally, both infant donors and colonized mice exhibited different bacterial composition between healthy and cow’s milk allergic groups (Feehley et al., 2019). In another study, *Clostridia* enrichment of gut microbiota was found to be able to protect against sensitization to food allergen, suggesting its role in food allergy prevention. The protection mechanism include regulation of innate immune system as well as the intestinal epithelial permeability (Stefka et al., 2014). Besides, colonization of mice by a mixture of *Clostridium* strains was shown to suppress systemic immunoglobulin E responses (Atarashi et al., 2011).

One breakthrough of the research in this area is to clarify the critical role of timing. In addition to display the link between the richness of gut microbiota and *Enterobacteriaceae/Bacteroidaceae* ratio at 3 months was linked with food sensitization at 12 months, the Canadian researchers also determined that the intestinal bacterial profile in infants at 12 months had no association to food sensitization (Azad et al., 2015). A study based on murine model provided further evidence for the early-life microbial exposure. Gnotobiotic mice and those with low-diversity microbiota developed abnormally high level of serum IgE in early life. Then the investigators exposed the germ-free mice to different numbers of bacterial species at different life time. The mice in the group that colonized with microbiota consisting of 40 phylotypes early, but not late, in life were protected from food allergy and showed undetectable serum IgE level (Cahenzli et al., 2013).

These findings further propelled our understanding of microbial effect on food allergy in that early infancy is an important window during which the composition of intestinal bacterial may manipulate subsequent food allergy development.

## Respiratory Allergy and Gut Microbiota

Aside from food allergy, respiratory allergy has also been linked to gut microbiota dysbiosis. The most typical respiratory allergic disease is allergic rhinitis (AR), which is part of a systemic airway inflammatory disorder and affects people of all ages. According to epidemiologic studies, 20–30% of adults and around 40% of children have suffered from AR (Greiner et al., 2011; Kakli and Riley, 2016; Hoyte and Nelson, 2018). AR is not a dreadful illness on itself, however its symptoms meddle in all facets of daily life, leading to reduced sleep quality and work performance. It is also a common reason for clinical visits in general practice, which could trigger many complications and comorbid with asthma (Kakli and Riley, 2016; Brożek et al., 2017). This loss of performance, missed work days, and the expense for AR treatment have created heavy cost to both individual and society. Swedish researchers have reported that the cost for AR is € 2.7 billion per year nationally (Hellgren et al., 2010).

Asthma is another very common airway allergic disease. Together with allergic rhinitis, they constitute the most important health problem in children (Schatz and Rosenwasser, 2014; Chiu et al., 2019). It is reported that about 300 million people suffered from asthma across the world and the figure would go up to 400 million by 2025 (Dharmage et al., 2019). Asthma and allergic rhinitis often happen together. The economic cost for asthma is also huge. In the United States alone, the annual cost (including both medical expenses and absence from work/school) reached 81.9 billion (Nurmagambetov et al., 2018). Epidemiological data suggest that most asthma patients have concomitant AR and the occurrence of AR increase the risk for asthma development (Khan, 2014).

The critical role of GI microbiome in airway allergic disorders has been proved by a tremendous number of epidemiological and microbiological studies in the last decade. Murine studies showed that intestinal epithelial cell (IEC) was able to shape the microbiota residing in GT and further to influence the immune react in the airway. The deletion of IKKβ signaling in IECs could significantly elevate the percentage of *Clostridium* species and segmented filamentous bacteria, a bacterium that is similar with *Clostridium* in terms of 16S rRNA. These two bacteria could support Th17 and IgA Ab responses (Bonnegarde-Bernard et al., 2014). Studies with germ-free (GF) mice indicated a direct link between GI microbiome and airway inflammation. Compared to specific pathogen–free (SPF) group, the GF mice with allergic airway inflammation presented elevated number of infiltrating lymphocytes and eosinophils. And this evaluation could be restored by recolonization of feces from SPF mice to GF mice (Herbst et al., 2011). Unlike high-fat diet, sufficient intake of dietary high fiber was testified to strongly inhibit OVA-induced allergic rhinitis. Enriched levels of Bacteroidetes and Actinobacteria, decreased proportion of Firmicutes and Proteobacteria might contribute to this protection effect (Zhang et al., 2016). Mice treated with *Bifidobacterium longum* IM55 and *Lactobacillus plantarum* IM76 reduced the level of serum IgE as well as OVA-induced IL-4 and IL-5 levels in nasal tissues. The disturbed gut microbiota caused by OVA was also restored by these two bacteria strains (Seo et al., 2015). A mixture of *Bifidobacterium breve* and non-digestible oligosaccharides was also able to inhibit pulmonary airway inflammation possibly by the modulation of regulatory T cell response (Sagar et al., 2014).

These connections are also supported by clinical studies. Numerous studies have noticed that children raised up in rural regions or conventional farms showed a lower incidence of asthma and allergic rhinitis onset (Douwes et al., 2008; von Mutius and Vercelli, 2010; Jie et al., 2013; Rennie et al., 2016). This phenomenon should be attributed to the frequent contact with animals and/or grains, the environment of which is more microbial-diverse (Douwes et al., 2008; Ege et al., 2012). Ege *et al.* compared the microbial data of 489 school aged children from rural and urban areas in Germany, and identified a number of bacteria, such as *Acinetobacter*, *Lactobacillus*, and *Staphylococcus* which were inversely related to asthma and hay fever (Ege et al., 2012). When comparison was made between children from similar surrounding environment, *e.g.*, both from urban region, a mild reduction in terms of microbiota diversity was measured in the children with allergic airway diseases and the microorganisms from phylum Firmicutes were significantly less expressed than healthy children (Chiu et al., 2019). This similar trend was also observed with Swedish children. Children with asthma possess a lower diversity of gut micro-bacteria compared to children without asthma at infancy (Abrahamsson et al., 2014). Demirci *et al.* quantified the amount of *Faecalibacterium prausnitzii* and *Akkermansia muciniphila* which could induce the secretion of anti-inflammatory cytokine whilst suppress the production of pro-inflammatory cytokines. Both of them reduced significantly in the fecal samples of allergic asthma children (Demirci et al., 2019). Another study conducted on 1,228 siblings with or without asthma revealed that the use of antibiotics of mums during month 7 to month 9 of pregnancy could enhance the susceptibility to asthma in preschool children (Mulder et al., 2016). Interestingly, early exposure (within the first 12 months after birth) to antibiotics was only strongly associated with the onset of asthma only, not allergic rhinitis. But, allergic rhinitis was related to lifetime usage of antibiotics (Ni et al., 2019). Consumption of combined *L. gasseri* TMC0356 (TMC0356) and *Lactobacillus rhamnosus* GG (ATCC53103) contained in cultured dairy product for around 3 months was able to modify the composition of gut microbiota in Japanese cedar allergy patients (Harata et al., 2017). All the studies demonstrated the correlation between intestinal bacteria and airway allergic disease.

## Skin Allergy and Gut Microbiota

Skin diseases ranked the fourth primary cause of non-fatal illness burden worldwide. Among them atopic dermatitis is the leading one triggered by cutaneous hyperactivity to environmental allergens (Leung et al., 2004; Hay et al., 2014; Lopez Carrera et al., 2019). Atopic dermatitis, characterized by prominent skin itching and relapsing eczematous lesions, is a chronic skin inflammatory disorder which could further result in allergic rhinitis, asthma, and food allergy (Weidinger and Novak, 2016). It usually starts in infancy and children; thus atopic dermatitis was originally regarded as a pediatric disorder. But now increasingly research indicates that atopic dermatitis is also a common disease in adults (Lopez Carrera et al., 2019; Silverberg, 2020). It is estimated that in many affluent country settings, atopic dermatitis affects more than 20% children and the onset of atopic dermatitis presenting within the first 2 years of life in children is 21.5% (Thomas and Fernandez-Penas, 2017; Sugita and Akdis, 2019). A British population survey found that among 1,760 affected children, 84% of the children exhibited mild symptoms, 2% showed severe atopic dermatitis, and the rest were mild (Williams, 2005). Epidemiologic data on adult indicate that 3% of the global adults are suffering from atopic dermatitis and a greater incidence was observed among Asian populations (Mei-Yen Yong and Tay, 2017; Lopez Carrera et al., 2019). The prevalence of atopic dermatitis in different racial and ethnical groups is still rising, including Western and Northern Europe, Africa, and Eastern Asia (Deckers et al., 2012; Flohr and Mann, 2014).

Like respiratory allergy, atopic dermatitis also imposes a huge economic burden to the society. Cost of illness study indicated that the average cost for atopic dermatitis varied from US$71 in Netherland to US$2,559 in Germany *per capita* annually (Verboom et al., 2002). Atopic dermatitis is a complex disease, involving multiple factors which include genetic factors, a defective barrier, environmental factors, and immunologic responses (David Boothe et al., 2017). Discovery of inactivating mutations in filaggrin protein has shed new light on atopic dermatitis mechanism, which has been implicated in sever atopic dermatitis pathology (O’Regan et al., 2008; David Boothe et al., 2017). As interface organs, the skin shares a number of common features with the gut, for example being integrated into the overall immune system. Thus, it makes sense to have skin co-morbidities with gut disorders (O’Neill et al., 2016). The recognition of gut and skin connections could date back to 1930s when Dr. Donald M. Pillsbury and Dr. John H. Stokes, the two most influential dermatologists in the last century, came up with the idea of a gastrointestinal mechanism for skin ailments like acne (Stokes and Pillsbury, 1930; Bowe and Logan, 2011). In their theory, altered intestinal microflora induced by stress caused skin inflammation (Bowe and Logan, 2011). Then entering 21^st^ century, many aspects of this “gut-skin axis” have been validated with both murine and human studies. A pile of cohort studies has implied that lower diversity of intestinal microbe in infants precede atopic dermatitis development in the later life. The intestinal dysbiosis started to occur in infants with eczema at day 7 which was proven to be inversely correlated with the severity of eczema. A reduced multiplicity of the phylum Bacteroidetes and phylum Proteobacteria were observed in the fecal samples at the first month and twelfth month of newborns who had IgE-mediated eczema (Abrahamsson et al., 2012; Ismail et al., 2012; Nylund et al., 2015). US infants who are more prone to have atopic dermatitis and asthma in later life demonstrated decreased relative abundances of *Bifidobacterium*, *Akkermansia*, and *Faecalibacterium* (Fujimura et al., 2016). Moreover, *Coprococcus eutactus*, a butyrate producer, was in a lower quantity in atopic dermatitis infants whilst *Clostridia* was more abundant in atopic dermatitis infants (Nylund et al., 2015; Lee et al., 2016). Infants colonized with *Clostridia* at week 5 and 13 after birth were significantly correlated with the onset of atopic dermatitis in the subsequent 6 months (Penders et al., 2013). Enrichment of *Faecalibacterium prausnitzii* in patients’ fecal samples was shown to be strongly associated with atopic dermatitis (Song et al., 2016). Another report indicated that *Faecalibacterium prausnitzii*, together with *Escherichia coli*, *Bifidobacterium adolescentis*, and *Akkermansia muciniphila* were highly discriminant for atopic dermatitis teenagers whether having food allergy or not (Fieten et al., 2018). In animal study, atopic dermatitis mice administered with antibiotics exhibited intensified symptoms with increased level of IgE and interleukin 4 in comparison with those having received probiotics or feces from healthy mice (Kim et al., 2020).

In turn, certain bacteria strains can alleviate atopic dermatitis. Nickel allergy is the most common contact dermatitis caused by direct contact with nickel with both skin and GI symptoms (Braga et al., 2013). A double-blind randomized placebo controlled study showed that treatment with probiotic *Lactobacillus reuteri* strain can reduced GI and cutaneous symptoms of nickel allergy patients (Randazzo et al., 2015). In a more recent study, *Lactobacilli*- or *Bifidobacteria*-containing formulation could not only ameliorate Ni-sensitivity, but also restore the gut dysbiosis detected in patients (Lombardi et al., 2020). *Bifidobacteria adolescentis* was able to ameliorate DNFB-induced atopic dermatitis in rodent by promoting Treg differentiation and suppressing Th2 response (Fang et al., 2019). Erdman’s group found that the mice consuming probiotic yogurt developed more lustrous fur with increased thickness of dermis, and upregulated folliculogenesis. In female mice, the pH of skin was also more acidic in the group eating probiotic yogurt and acidic pH is known to contribute to glowing fur (Levkovich et al., 2013). A pilot study with 109 atopic dermatitis patients revealed that *Lactobacillus plantarum* altered the alpha diversity of gut microbiota in atopic dermatitis patients and exerted amelioration effect of atopic dermatitis. *Lactobacillus plantarum* treatment brought down the SCORAD index, and increased IL-10 level in serum (Fang et al., 2019). Supplementary with *Bifidobacterium* LKM512 improved itch in atopic dermatitis patients and upregulated the level of kynurenic acid which is an antipruritic and metabolite (Matsumoto et al., 2014). To sum up, these studies support the novel concept that gut flora is a main regulator of “gut-skin” axis, the composition of which contributes to the onset of atopic dermatitis (Salem et al., 2018).

## Factors Affecting Gut Microbiota

### Genetic Factor

The influence of host genotype on determining the gut bacteria has only recently been recognized. The classic method applied to study the genetic factors is to compare the data between monozygotic (MZ) and dizygotic (DZ) twins (Kurilshikov et al., 2017). A large cohort study with samples from the TwinsUK population (n = 416) unveiled that monozygotic twins had more similar gut microbiota composition compared to dizygotic twins, proving the role of genetic factor in shaping the intestinal microbiome. This study also identified a number of heritable bacterial taxa, and the most heritable one belongs to the family of Christensenellaceae (Goodrich et al., 2014). Then 2 years later, the sample size was tripled by the same research group with 1,126 twin pairs. This enlarged sampling first confirmed inherited tax discovered before and uncovered new associations between host genes and bacterial strains (Goodrich et al., 2016). Several studies have also noticed a link between host specific genetic variants and individual bacterial strain. Turpin et al. reported 58 single nucleotide polymorphisms correlate to the relative levels of 33 bacterial strains. Of them, four loci were repeated in another cohort study (Turpin et al., 2016). With genome-wide analysis, Bleckhman et al. verified the relationship between host genotype and intestinal bacterial composition (Blekhman et al., 2015).

Although host genotype proved to be able to modulate the gut microbiota structure, it is not the key factor and its determination ability on intestinal bacteria constitution is not remarkable. A recent study indicated that 42 single nucleotide polymorphisms together were able to explain only 10% of the total variance in gut microbiota (Wang et al., 2016). A significant similarity in microbiome composition of genetically unrelated individuals who live together was observed, and at least 20% of the inter-personal microbial difference is the result of different diet habit, and/or drugs administration (Rothschild et al., 2018). This study also revealed that the establishment of gut microbiota is chiefly influenced by environmental factors, not genetic factor by analyzing the data from 1,046 healthy Israeli people (Rothschild et al., 2018).

### Perinatal Factors

Studies on germ-free mice suggested that microbial exposure at early life is critical for normal immune system development which is necessary to control baseline IgE level (Cahenzli et al., 2013). It was once universally accepted that fetal GI tract was sterile with first microbial colonization starting at delivery (RodrÍguez et al., 2015). However, this dogma is being challenged by the discovery of low abundance microbes in placenta, meconium, and amniotic fluid (RodrÍguez et al., 2015; Nyangahu et al., 2018). The advent of molecular techniques such as next-generation sequencing, has allowed scientists to conduct microbiology research on womb. The research that employed these approaches demonstrated that microbes are presenting in the fetal environment with the characters of low richness and low diversity, and proposed that the colonization of intestinal microbiota in infants already initiated before birth (Jones et al., 2009; Stout et al., 2013; Collado et al., 2016; Perez-Muñoz et al., 2017). In a small study (n = 29), microorganisms were detected in all of the 29 placental samples with *Lactobacillus* being the predominant one, followed by *Bifidobacterium* (Rautava et al., 2012). An extended study carried out with 320 subjects in a case-cohort design described a unique microbiome signature in the placenta, which include Tenericutes, Firmicutes, Bacteroidetes Fusobacteria, and Proteobacteria phyla with the last one being the predominant one. By comparing to other niches of human body, the micro-bacterial profile of placenta is most akin to the microbiome residing in oral cavity (Aagaard et al., 2014; Collado et al., 2016).

#### Mother’s Nutrition

The mother’s nutrition during pregnancy is a key factor affecting the baby’s microbiome. An under-nutrition diet of pregnant mothers could result in the establishment of inadequate intestinal microbiome of infants (Salazar et al., 2014). Another study showed that maternal consumption of salmon tended to increase the prevalence of Atopobium cluster in the offspring (Urwin et al., 2013). The intake of probiotics by mothers during pregnancy alters the gut microbiota composition of infants. For example, neonates whose mothers took *L. rhamnosus* GG presented a higher bacterial counts of *B. breve* and a declined relative abundance of *B. adolescentis* compared to the infants whose mothers received placebo (Sanz, 2011). Besides, the pregnant women with inflammatory bowel disease would pass on altered bacteria to their babies with lower diversity than healthy mothers (Torres et al., 2020). In addition, investigators pointed out that gestation time might also affect the colonization of gut microbiota. Preterm infants had lower diversity of cultural bacteria compared to full-term infants and characterized by *Lactobacillus*, *Streptococcus*, and *Carnobacterium* in later life (Rouge et al., 2010; Storrø et al., 2011; Fouhy et al., 2019).

#### Delivery Type of Infants

It is not astounding that delivery mode creates great differences regarding to intestinal bacteria composition in offspring due to the fact that cesarean section delivery could possibly disrupt the bacterial transmission from mothers to newborns owing to the lack of exposure to the maternal vaginal bacteria (Mueller et al., 2015; Blaser and Dominguez-Bello, 2016; Kim et al., 2019). As expected, babies delivered by caesarean section exhibited a disrupted transmission of *Bacteroides* from mother as well as enriched establishment of opportunistic pathogens (Shao et al., 2019). Besides, standard vaginally delivered neonates colonized more microbes from vaginal microbiota of their own mothers’ whist infants born with cesarean are typically harbored greater numbers of bacteria from skin surface or environmental origins (Dominguez-Bello et al., 2010; Shao et al., 2019). A study involved 108 healthy newborns demonstrated that the establishment of *Bifidobacterium*, *Lactobacillus*, and *Bacteroides* were influenced most profoundly by the delivery mode (Martin et al., 2016). C-section delivered infants acquired lower levels of *Bifidobacterium*, *Clostridium*, *Enterococcus*, *Klebsiella* spp., and *Parabacteroides* species, but enriched with *Staphylococcus*, *Corynebacterium*, and *Propionibacterium* spp. compared to standard vaginally delivered babies (Dominguez-Bello et al., 2010; Mueller et al., 2017; Liu et al., 2019; Reyman et al., 2019). Furthermore, babies born by C-section have a postponed colonization of *Bifidobacterium* and *Collinsella* with low diversity and richness of species, and exhibited less resemblance to their mums (Azad et al., 2013a; Backhed et al., 2015; Dogra et al., 2015). A newly published research with 440 children also showed that the enrichment of Bacteroides species from week 5 to week 31 was greatly associated with vaginal delivery (Galazzo et al., 2020). These changes in gut microbiota composition as a result of different delivery modes have been linked to metabolic and immune system disorders, especially the immunological functions of the infants during the first year of life (Huurre et al., 2008; Kim et al., 2019). To normalize the intestinal bacteria, Domingues-Bello *et al.* swabbed the babies delivered by C-section with their mothers’ vaginal fluids. In the first month, they presented similar bacterial communities to vaginally delivered infants, but the long-term benefits still require investigations (Dominguez-Bello et al., 2016).

#### Breastfeeding

It is well-known that breastfeeding could provide both short-term and long-term benefits for children development, hence the World Health Organization proposed an exclusively breastfeeding strategy for the first 6 months (Moore and Townsend, 2019). Recent investigations revealed that breast milk contains an array of bacteria, dominated by *Bifidobacteria*, *Streptococci*, *Lactobacillus*, and *Staphylococci*, making it an ideal supply of commensal bacteria to neonate intestine and another important factor that significantly influence microbiome colonization (Hunt et al., 2011; Fernandez et al., 2013). By exposing the infants to breast milk, the “milk microbiome” could be transferred to babies directly and help the colonization of microbes in the infant gut (Hunt et al., 2011; Jeurink et al., 2012). Compared to formula-fed newborns, the gut of breast-fed neonates was inhabited with more complex *Bifidobacterium* species, and increased relative abundance of *Lactobacilli* and *Bifidobacteria* (Roger et al., 2010; Pascal et al., 2018). Interestingly, a metagenomics study performed on 98 fecal samples from Swedish newborns indicated that termination of breastfeeding is a chief driver towards the development of adult-like microbial community. The gut of non-breastfed infants at 1 year old was enriched with *Clostridia* that are ubiquitous in grown-up persons, but the intestinal microbiota of infants who still receive breast milk at the age of 12 months was dominated by *Bifidobacteria* and *Lactobacillus* (Backhed et al., 2015). However, in another study, Galazzo et al. observed a decreased levels of *Bifidobacteria*, *Staphylococci*, and *Streptococci* upon cessation of breast milk (Galazzo et al., 2020). Moreover, breastfeeding was able to partially restore the gut microbiota disturbance caused by C-section delivery and increase the richness as well as microbial diversity of preterm infant (Liu et al., 2019; Zanella et al., 2019).

In addition to the bacteria, breast milk contains a range of bioactive components that facilitate the healthy establishment and maturation of newborn’s gut microbiota (Ballard and Morrow, 2013). For example, research indicated that breast milk-derived secretory IgA was able to promote a healthy gut microbiota composition and prevent the translocation of opportunistic pathogen (Rogier et al., 2014; van den Elsen et al., 2019). Oligosaccharides, as the third most abundant component in the human milk, were also proven to be able to propel the growth of “healthy” microbiota, such as *Bifidobacteria* (Zivkovic et al., 2011; Marcobal and Sonnenburg, 2012; Charbonneau et al., 2016). They can act as receptor analogs to prevent pathogens’ attachment to the intestinal surface. Moreover, non-digestible oligosaccharides could be fermented into short chain fatty acids which propel the proliferation of probiotics (Walker, 2010).

### Exposure to Antibiotics

The effect of antibiotics on gut microbiota of human have been extensively investigated ever since the discovery of antibiotics (Modi et al., 2014). Antibiotics could reduce the diversity of microbiome, for example macrolide was shown to inhibit the growth of *Bifidobacterium*, *Bacteroides*, *Collinsella*, *Lactobacillus*, and *Anaerostipes* (Korpela et al., 2016).

In the US, it is estimated that more than half of the pregnant women are taking antibiotics during pregnancy (Blaser and Dominguez-Bello, 2016). Antibiotics intake during pregnancy has been shown to alter the microbiome of the birth canal, which, in turn, leads to a modified commensal microbiota of infants (Stokholm et al., 2014). A longitudinal cohort study showed that there was a delay in terms of *Bifidobacterium* expansion, which is important in the infant and a persistence of *Escherichia* in the intestine of infants exposed to intrapartum antibiotics treatment (Stearns et al., 2017). In another study, a similar reduction in the abundance of *Bifidobacterium* species was noticed as well in the gut of newborns whose mothers took antibiotics during the labor. Besides, these newborns also had a declined quantity of *Actinobacteria* and *Bacteroidetes* (Aloisio et al., 2016). The prenatal exposure to antibiotics can also affect the later bacterial colonization of infants. One report demonstrated that at the age of 3 months, 13 bacterial strains presented at different levels between the infants with and without exposure to antibiotics in second trimester. The number of changed bacteria went up to 17 at the age of 12 months (Zhang M. et al., 2019). In a pilot study with 15 preterm infants, both short-term (≤3 days) and long-term (≥5 days) antibiotics intervention lowered the relative quantity of *Bifidobacterium.* It was found that antibiotics tended to inhibit the growth of Enterobacteriaceae family while promote Enterococcus to thrive (Zwittink et al., 2018). As for adult, short-term use of antibiotics is able to cause a profound fluctuation among the gut microbiota community as well. Studies revealed that 7-day course of antibiotics treatment decreased the overall diversity by 25% while elevated some unidentified taxa belonging to Bacteroides (Panda et al., 2014). Andrés Moya *et al.* monitored the change of gut microbial community of an individual subject to antibiotics for 14 days. The loss of Gram-negative micro-bacteria occurred on day 6 and the maximum imbalance in gut microbiota composition happened on day 14. The gut microbiota community reached the lowest diversity and richness on day 11. This study strongly confirmed the effect of antibiotic on gut microbiota (Pérez-Cobas et al., 2013).

### Living Style

#### Exposure to Pets

Microbial composition of humans, especially the infants, has been shown to be profoundly affected by indoor furry pets and early contact with these pets could lower the risk for allergic disease (Nermes et al., 2013; Tun et al., 2017). The furry pets could increase the diversity and richness of bacterial community of household dust, then leading to an altered microbial composition in the gut (Fujimura et al., 2010). The enrichment of *Ruminococcus* and *Oscillospira* was linked with exposure to pets, and negatively correlated to childhood atopy (Tun et al., 2017). There are also some bacterial strains reduced by pet exposure. For example, the bacterial strain of *Lactobacillus reuteri* was found to present at a lower proportion in the gut of infants from pet-keeping families than from the family without pets (Tun et al., 2017). A pilot study performed by Nermes *et al.* confirmed a direct transfer of bacteria from pets to babies. In their research, more *Bifidobacterium pseudolongum* and *Bifidobacterium thermophilum*, the two known pets-derived bacteria were measured in the feces of pet-exposed infants (Nermes et al., 2015). However, in contrast to this study, Azad et al. spotted an under-representation of *Bifidobacteriaceae* in newborns residing with pets (Azad et al., 2013b). Additionally*, Leifsonia*, *Exiguobacterium*, *Agrococcus*, *Herbaspirillum*, *Carnobacterium*, and *Neisseria* were found by a Canadian research group to be associated with dogs companion and *Escherichia* was indicative of exposure to cats (Konya et al., 2014).

#### Habitual Diet

Diet is another most obvious determinant of human microbiome diversity. It is reported that diet contributes to 57% of intestinal bacterial variations (Zhang et al., 2010). Human gut microbiota respond quickly to the change of diet (Dong and Gupta, 2019). It only took 1 day for an animal-based diet to increase the β diversity of healthy volunteers’ gut microbiota and this change could be restored within 2 days after this animal-based diet ended (David et al., 2014).

The existence or disappearance of several bacterial strains have already been linked to different diet styles. For instance, *Roseburia*, *Lachnospira*, and *Prevotella* were strongly correlated to vegetable-based diet. Notably, both *Roseburia* and *Lachnosipira* participate in the polysaccharides degradation to short chain fatty acids (SCFAs) (De Filippis et al., 2016; Barrett et al., 2018). Mediterranean diet (MD) is the most well-known diet pattern due to the health benefits it has brought to humans. It consists of high intake of whole grains, olive oil, fruits, vegetables, and nuts; medium intake of fish, chicken, and dairy products; low intake of red meat (Mitsou et al., 2017; Luisi et al., 2019). People who adhere to Mediterranean diet had higher *Candida albicans*, *bifidobacterial*, and *saccharolytic* species counts and lower prevalence of *Escherichia coli.* Besides, the olive oil in Mediterranean diet can induce the growth of lactic acid bacteria in the human gut (Mitsou et al., 2017; Garcia-Mantrana et al., 2018; Luisi et al., 2019). Furthermore, higher ratio of *Firmicutes* to *Bacteroidetes* and lower levels of short chain fatty acids were associated with less adherence to the Mediterranean diet (Garcia-Mantrana et al., 2018). Studies revealed that Mediterranean diet is good for amelioration of obesity and inflammation which might be intervened by these diet-derived bacterial strains (Singh et al., 2017). In contrast to Mediterranean diet, a Western diet, which is high in animal fat, simple carbohydrate, and low in fiber, is regarded as a non-healthy balanced diet. Evidence of the impact of Western diet on host gut microbiota came from studies in germ-free mice. Siddharth *et al.* compared the microbial profile of mice fed with Western diet and normal mouse chow. *Marvinbryantia*, *Clostridium* XVIII, and *Alistipes* were found to be connected to the Western style diet (Siddharth et al., 2013). Another study demonstrated that Western diet could create an environment for inflammation characterized by the overgrowth of *Escherichia coli* and inhibition of protective bacteria (Agus et al., 2016). It is noteworthy that this inhibition of intestinal bacteria by Western diet could lead to final extinctions of these taxa over generations, and the reconstruction of gut bacteria to its original condition requires administration of both missing species and dietary fiber (Sonnenburg et al., 2016). A comparison made between European children and rural African children’s fecal samples further confirmed the role of Western diet on microbiome. The gut of African children inhabited more *Bacteroidetes* and depleted *Firmicutes* (De Filippo et al., 2010).

Research on the effect of specific dietary component on intestinal bacteria has also been carried out. Noriega *et al*. explored the influence of fatty acids on gut microbiota. Fourteen days later*, Faecalibacterium prausnitzii* and *Akkermansia* decreased greatly whilst several butyrate-producing bacterial strains increased (Noriega et al., 2016). Dietary fiber (DF) was considered to generate beneficial effect on gut microbiota. It has been shown to alter the ratio of *Firmicutes* to *Bacteroidetes* (Trompette et al., 2014). DF with discrete chemical structures presented distinct microbiome manipulation ability. For example, Deehan et al. compared different type-IV resistant starches (RS4s) and found that maize RS4 precisely stimulates the reproduction of *Eubacterium rectate* which could produce butyrate whilst tapioca RS4 specifically promotes the growth of propionate-generating taxa, namely *Parabacteroides distasonis.* Meanwhile, potato RS4 had no effect on microbiome (Deehan et al., 2020). DF enriched with arabinoxylan-oligosaccharides is able to boost the growth of *Prevotella* species (Benítez-Páez et al., 2019).

## Techniques Utilized in Microbial Analysis

### Microbial Culture Techniques

Bacterial culture was the first technique applied in the analysis of mammalian gut microbiota and used to be the only choice (Lagier et al., 2015b). Although it was once considered as outdated by many microbiologists, it is still an essential tool to explore the characteristics of bacterial colonies, and to assess the biological effect of a microorganism to human body (Lagier et al., 2015a). According to Robert Koch’s postulates, the isolation and cultivation of a single bacterium is the prerequisite to establish a link between bacteria and diseases (Segre, 2013). A pure culture also makes it possible to add or delete genes of a specific bacterial to analyze its invasive ability or boost its beneficial effect (Lagier et al., 2012; Piñero-Lambea et al., 2015). Culture media can be classified as non-selective culture media and selective culture media. In non-selective culture media, there is no inhibitors that permits the growth of the majority of microbes while selective culture media is added with inhibitors, in which only specific bacterial strains survive (Lagier et al., 2015a).

In addition to cultivate a single bacterial strain, two or more different populations of microorganisms can be grown together with some degree of contact among them, which is termed as co-culture system (Goers et al., 2014). This co-culture system is fundamental in demining microbe-microbe interactions including both antagonism and mutualism, as well as their response to perturbations (Mitri and Foster, 2013). In recent years, this system has been particularly employed in microbial chemical biosynthesis, to make use of the interactions among different bacterial strains (Mitri and Foster, 2013). An example in case is the co-cultivation of *Escherichia coli* and *Saccharomyces cerevisiae* to produce paclitaxel precursor. In this research, *Escherichia coli* is responsible for the *de novo* biosynthesis of the intermediate taxadiene, yet, *Escherichia coli* is not able to express cytochrome P450 which is compulsory for the following monooxygenase-catalyzed bioconversions. *Saccharomyces cerevisiae* can compensate for this by generating enough cytochrome P450. By combining them together, the authors could obtain 33 mg/L oxygenated taxanes (Zhou et al., 2015). Das *et al.* co-cultured *Bifidobacterium adolescentis* and *Bacteroides thetaiotaomicron*. Data revealed that *Bifidobacterium adolescentis* and *Bacteroides thetaiotaomicron* could compete for nutrient in the media with a higher level of *Bifidobacterium adolescentis*. They also addressed that these two strains cannot grow together because of environmental pH changes driven by short chain fatty acids (Das et al., 2018).

However, the drawback of this technique lies in that it is mainly based on pure culture technology, which is time-consuming, labor-intensive, and complicated. It is greatly restricted by the culture conditions, leading to the instability of the research results.

### Next Generation Sequencing Techniques

The emergence of high-throughput sequencing techniques together with diverse bioinformatics methods has boosted the research on human microbiome greatly, unveiling the mask of microbiome (Schmidt et al., 2018; Cheng et al., 2019). Compared with bacterial culture techniques, next generation sequencing (NGS) technology can be used to obtain information on all species and even their genomes, breaking through the limitations of microbial culture technology. At present, there are two main types of NGS sequencing used in intestinal bacteria research: 16S ribosomal RNA (16S rRNA) gene sequencing and metagenomics sequencing.

16S ribosomal RNA gene sequencing is by far the most extensively used sequence-base techniques for bacterial taxonomy and phylogeny study (Malla et al., 2019). It involves sequencing designated microbial amplicon, principally 16S ribosomal RNA, and the sample processing met (*e.g.* DNA extraction) has a noteworthy impact on the sequencing data (Franzosa et al., 2015; Walker et al., 2015). The 16S rRNA gene is present in all micro-bacteria, which codes for the RNA component of the 30S small subunit of the bacterial ribosome, and the 16S variable regions are bacterial specific (Wang et al., 2015; Malla et al., 2019). 16S rRNA gene sequencing has a great potential in identifying genus or species that don’t fit any documented biochemical profiles (Janda and Abbott, 2007). Although 16S rRNA gene sequencing is of significant use in terms of bacterial identification and classification, its low-resolution level restricts its application. Besides, 16S rRNA mainly gives information about “who are there,” in other words, it generally provides compositional information about microbiome, lacking functional annotation (Janda and Abbott, 2007; Cheng et al., 2019).

Shotgun metagenomics is a relatively new sequencing approach which advances our understanding of microbiota functions in the host (Malla et al., 2019). Shotgun metagenomic sequencing targets at the entire microbial genomes contained in a community (Quince et al., 2017). This approach can not only answer the question “ who are there,” but also “what they can do,” making itself a powerful technique (Franzosa et al., 2015). Shotgun metagenomic sequencing was first employed to study the microbial diversity of ocean. Later in 2006, Gill *et al.* performed metagenomics on healthy human genome, describing the genome profile and encoded function ascribed to gut bacteria, which improved our understanding of microbial diversity (Gill et al., 2006). From then on, shotgun metagenomics sequencing has been implicated in numerous large-scale studies in different aspects of this complicated microbial community (Quince et al., 2017). European women suffered from type 2 diabetes displayed elevated abundance of Lactobacillales and alterations in Clostridiales bacterial counts with metagenomic sequencing (Karlsson et al., 2013). This approach also facilitated researchers to find a panel of microbiota-targed biomarkers for the diagnosis of liver cirrhosis with high accuracy (Qin et al., 2014). Armour *et al.* conducted the first met-analysis of 2,000 metagenomic samples spanning eight clinical studies and seven diseases, and they managed to define the characteristic bacterial profile of different diseases (Armour et al., 2019). However, metagenomics sequencing also has its own limitations. It might lose genomic information of some low abundance bacteria and it is unable to examine the functional attributes of microbiota directly under a given circumstance (Franzosa et al., 2015; Mende et al., 2016).

### Functional Omics Techniques

As is mentioned above, the fundamental drawback of current sequencing techniques is a lack of desired functional activity evaluation; while functional omics approaches can not only compensate for this deficiency but also are more variable and sensitive to perturbations compared to genomes (Franzosa et al., 2015; Heintz-Buschart and Wilmes, 2018).

**Figure 1:**
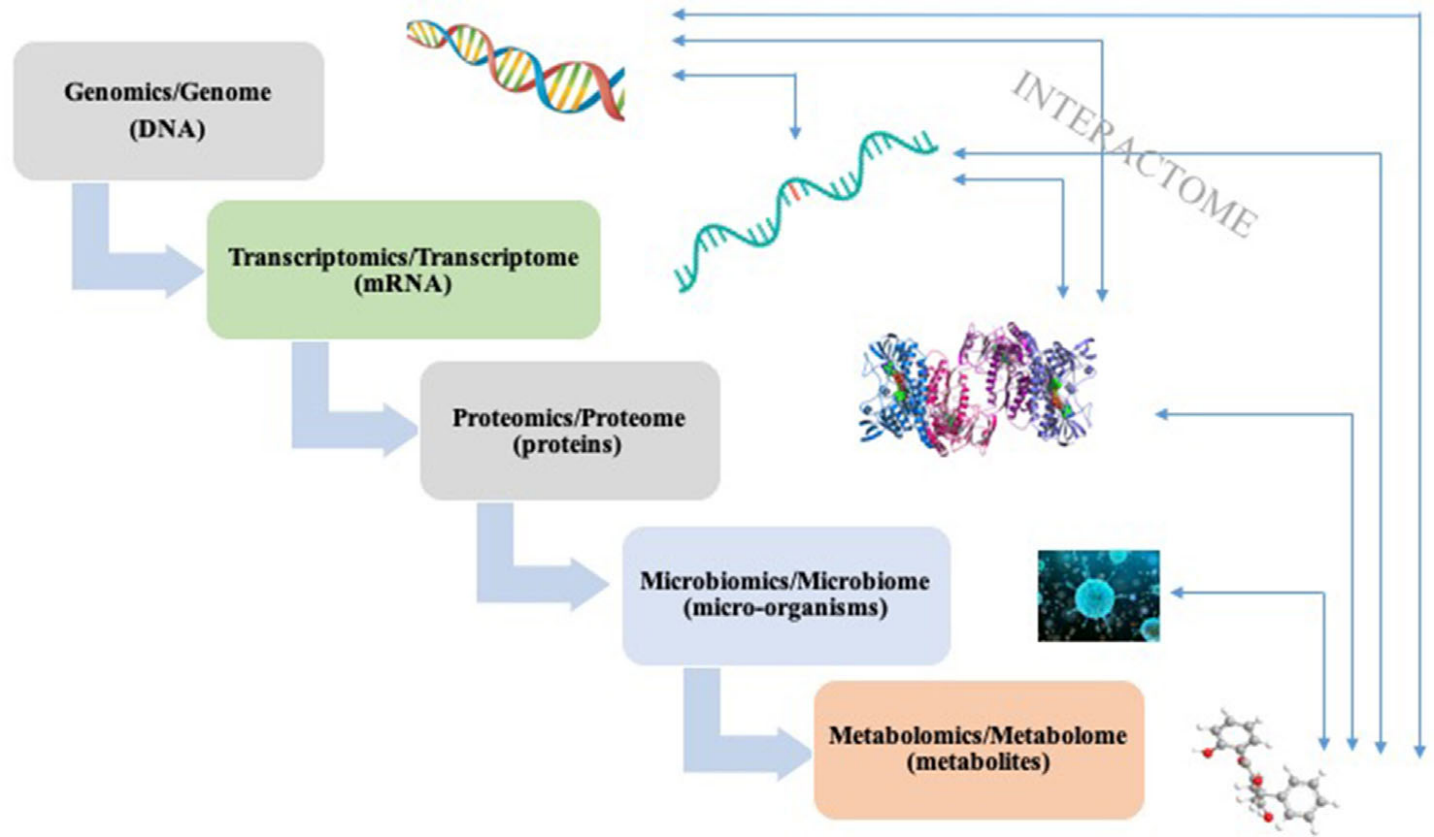
A simplified schema of “-omics” techniques and their corresponding analysis targets. Interactions can occur between different stages.

Functional omics techniques include transcriptomics, proteomics, and metabolomics, and their applications have successfully led remarkable progressions in linking gut microbiome with diseases (**Figure 1**) (Petriz and Franco, 2017). Transcriptomics is the measurement of entire RNA transcripts of a microbial community under specific conditions. By comparing transcriptome, we could pin-point the genes that are expressed differently (Heintz-Buschart and Wilmes, 2018). Pan *et al.* utilized epithelial transcriptome to examine the possible connections between gut microbiota and gene expression. Data revealed that the intestinal bacteria have a dramatic effect on transcriptional program of intestinal epithelial cells after birth (Pan et al., 2018). A combined transcriptomics and 16S rRNA gene sequencing indicated that with chronic ethanol consumption, the expression of genes involving inflammation, colorectal cancer (CRC) development, together with some microbes linked to inflammation and CRC changed greatly (Barr et al., 2018). Proteomics aims to measure the complete set of proteins in a system and it is proven to be a valuable approach in the investigation of the probiotic functionality of *Lactobacillus pentosus* strains (Ruiz et al., 2016). This approach was once used to analyze the adhesion ability of mucus of 31 *Lactobacillus pentosus* species, leading to the discovery of moonlighting protein that are associated with strain specific adhesive capability (Perez Montoro et al., 2018). Proteins expressed by human are of great importance in keeping the mutualistic relation between host and inhabited microorganisms (Lichtman et al., 2013). In a probiotic intervention trial, proteins from human origin represent 14% of the identified proteome and exhibited variations both between and within individual, suggesting a host-microbiome interaction (Kolmeder et al., 2016). Metabolomics is the youngest in functional omics techniques, which targets at all the metabolites. Metabolomics acts as an independent line of evidence for the hypothesis generated with sequencing techniques (Schmidt et al., 2018). For example, implication of metabolomics confirmed the role of intestinal microbiota metabolism on insulin sensitivity, and a link between gut microbiome and chronic kidney diseases (Aron-Wisnewsky and Clément, 2016; Kristensen et al., 2016).

Apart from the promising application of these –omics approaches, the important challenge lies there is the integration and interpretation of the obtain multi-omics data, which requires proper bioinformatics tools to mine the big data (Schmidt et al., 2018; Cheng et al., 2019).

### Genome Editing Techniques

**Figure 2:**
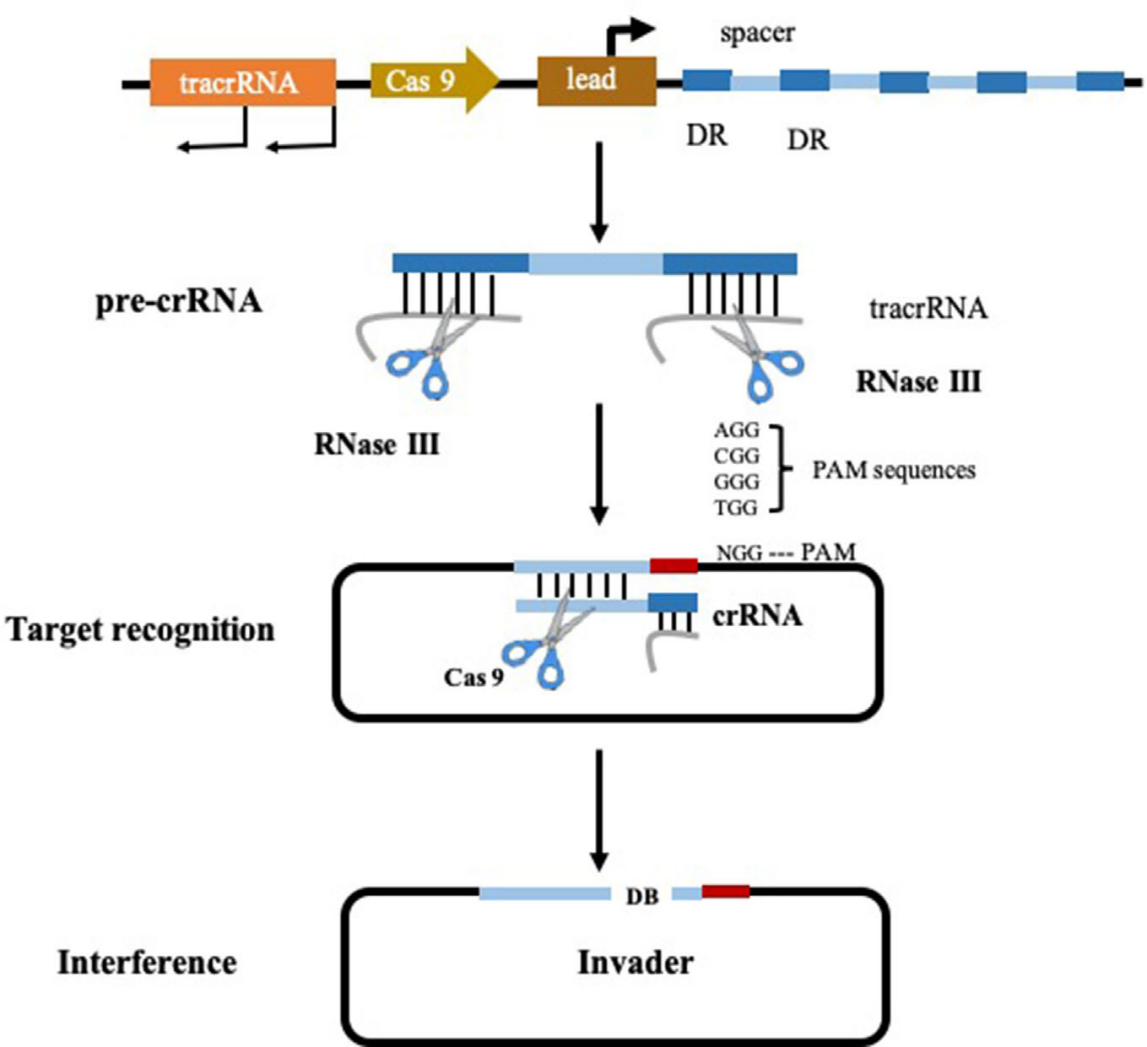
Type II CRISPR-Cas 9 system (Pyne et al., 2016).

With the in-depth study of the gene function of intestinal bacteria, researchers expect to use the genetic manipulation techniques commonly used by microorganisms to modify the genome of the intestinal bacteria to confer new functions or reduce toxicity. Clustered regularly interspaced palindromic repeats (CRISPR) and its associated proteins (Cas), have become a popular method due to the fact that it enables the targeted modification of specific gene sequencing (Jiang and Marraffini, 2015). The CRISPR/Cas systems can be categorized into type I, type II, and type III, of which type II is the most studied and utilized. The type II CRISPR system functions in a different mechanism from type I and III systems (type I and III systems share some common features) (Jinek et al., 2012). It is characterized by the presence of the RNA-guided endonuclease CRISPR associated protein 9 (Cas 9) (**Figure 2**) and this targeted genome editing technique intervened by Cas 9 is straightforward to engineer and scalable, allowing researchers to interpret the causal relationship between genotype and phenotype (Jiang and Marraffini, 2015). In 2013, the application of CRISPR-Cas 9 system reaches a peak time, resulting in considerable research outputs. Precise mutations in the genomes of *Escherichia coli* and *Streptococcus pneumoniae* have been achieved with CRISPR-Cas 9 system by researchers from Jiang’s group. In this pioneer study, mutation efficiency of *Escherichia coli* and *Streptococcus pneumoniae* were 65 and 100% respectively. This is the first reported application that CRISPR-Cas system was applied in the precise genome reformation in bacteria (Jiang et al., 2013; Ramachandran and Bikard, 2019).

At present, CRISPR-based gene editing technology has been successfully applied to a variety of intestinal microorganisms, such as *E. coli*, *Lactobacillus plantarum*, *Clostridium acetobutylicum*, *Clostridium beijerinckii*, *Staphylococcus aureus*, *Bacillus subtilis, etc.* (Ramachandran and Bikard, 2019). CRISPR-Cas gene editing technology is gradually becoming mature which acts as an exciting approach in the elucidation of bacterial roles within complex communities and drives the development of novel medication strategy.

### Mass Spectrometry (MS) and MS Coupled With Chromatography

Mass spectrometry (MS), including tandem MS, is another major technique to explore the biological function of gut microbiome from the perspective of gut microbiota derived metabolic profile (Zhang et al., 2012). The high sensitivity allows the detection and measurements of many gut microbial metabolites which are at low levels (Aretz and Meierhofer, 2016). Instead of being used alone, MS is often coupled with chromatographic technology. This approach combines the advantage of separation power of chromatographic technology with the high sensitivity and selectivity of MS. Separation prior to detection reduces the matrix effect and ion suppression that allows for a more accurate quantification and compound identification (Lei et al., 2011).

Gut microbiota-derived metabolites play a vital role in modulating human health, the analysis of which is indispensable to understand the underlying mechanism of the function of intestinal bacteria (Xu et al., 2019). SCFAs are the most widely studied metabolites produced by gut microbiota. Ma et al. established a sensitive liquid chromatography coupled to tandem mass spectrometry (LC-MS/MS) method with 2-bromoacetophenone as the derivatization reagent. This method improved the limit of detection of SCFAs greatly compared to conventional gas chromatography (GC), particularly for butyrate. With this method, the lower quantitative limit of butyrate can reach 1 ng/ml, 1,600-fold sensitive than conventional GC method (1.6 μg/ml) (Ma et al., 2019). At a similar time, Zhang et al. proposed a validated GC-MS method with silylation for the quantification of SCFAs in both feces and serum. In this method, sodium sulfate was applied to remove the moisture in SCFAs. Select ion monitoring mode was applied and it enabled the detection of SCFAs at the levels as low as 0.064 µM (Zhang S. et al., 2019). Bile acids (BAs) are another group of key metabolites that undergo microbial transformation. Both LC-MS and GC-MS have been employed to analyze BAs. Wegner et al. developed a rapid LC-MS/MS method to investigate microbial transformation of bile acids. With this method, they discovered the bile acids hydrolase activity of *Eggerthella lenta* and *Collinsella aerofaciens* (Wegner et al., 2017). Sánez et al. applied GC-MS to analysis the quantity of BAs in freshwater sediments (Sánez et al., 2017). More recently, Hu et al. established ultrahigh performance liquid chromatography–tandem mass spectrometry (UPLC-MS/MS) for a sensitive and efficient analysis of BAs in animals with *Tripterygium* glycoside-induced liver injury, and the results revealed a few BAs differ greatly between control and model groups (Hu et al., 2020).

## Intervention Strategy

In general, there is no cure for allergic disorders. The current management of allergies mainly includes allergen avoidance, medication treatment, and allergen immunotherapy.

### Allergen Avoidance

Allergen sensitization is essential in the development of allergic diseases. Hence identification and avoidance of the specific antigens should always be the first choice in allergy management (Kun and Li, 2016). This strategy is very straightforward. It has been promoted as a means to reduce the morbidity in the treatment of rhinitis and asthma and it is the current primary form for the treatment of food and insect sting allergies (Douglass and O’Hehir, 2006; Gold et al., 2017). Although this approach is widely recommended, it is not easily achieved in practice when applied to rhinitis and asthma. It is mainly due to the fact that people allergic to aeroallergens are often sensitized to multiple allergens, many of which cannot be determined by current methods. In addition, only 24% patients reported were willing to make changes which suggested to control the allergens exposure (Schatz and Zeiger, 2012).

### Medication Intervention

There are a number of over-the-counter or prescription medications in the form of tablets, eye drops, or nasal sprays, which could alleviate the symptoms caused by allergy. These drugs typically include antihistamines, decongestants, and corticosteroids. Among them, antihistamine and corticosteroid are gold standard remedies for allergic diseases treatment (Mayo Clinic, 2017).

Many allergic reactions are mediated by mediators secreted by mast cells. Histamine belongs to the most important mediators stored in mast cells. Upon stimulation, histamine will be released and act on the receptors located on endothelial cells, triggering a series of allergic reactions (Inagaki and Nagai, 2001). Antihistamines, also called histamine H1-receptor antagonists, aim to block the actions of histamine on H1-receptors for the purpose of diminishing or preventing the undesirable effects of histamine (Loew, 1947). It has been used for years and is the most widely applied drug to treat allergic symptoms. Antihistamines are effective to relieve the symptoms of running nose, red itchy eyes, and swelling. In addition to histamine, cytokines and leukotrienes are also involved in the allergic reactions, causing noticeable cellular infiltrate of lymphocytes, basophils, and eosinophils. This allergy-related inflammation can be suppressed by corticosteroids, making corticosteroids another widely employed drug for various allergic diseases (Kun and Li, 2016). Topical corticosteroids are indispensable for the treatment of dermatoses *via* binding to the glucocorticoid receptor. It is the first-line treatments for mild-to-moderate atopic dermatitis, which have shown satisfactory effect to control both acute and chronic cutaneous inflammation (Leung et al., 2004; Lopez Carrera et al., 2019). However, when it comes to severe atopic dermatitis, the treatment is not only costly, but also sometimes accompanied by adverse effect. For example, systemic corticosteroid therapy would lead to hyperglycemia, psychiatric disturbances, dyslipidemia, *et al.* (Liu et al., 2013; Lopez Carrera et al., 2019). Inhaled corticosteroids have been recommended as the first-line medications for persistent asthma as they can inhibit the production of chemokines, cytokines, as well as other adhesion molecules (Kun and Li, 2016; Mehta et al., 2016). Decongestants is applied to alleviate nasal congestion and are often prescribed along with antihistamines for allergic disorders. Decongestants are able to shrink blood vessels in the nose and open up nasal passages. Topical nasal decongestant is the most effect agent to act quickly on nasal symptoms, however, there is a high potential for overuse, resulting in side-effect such as elevated blood pressure (Green et al., 2020).

### Allergen Immunotherapy

For severe allergies or allergies that cannot be relieved by drugs, allergen immunotherapy might be recommended. Allergen immunotherapy is possibly the only medication approach that can modify the disease for allergic patients (Rodriguez-Dominguez et al., 2020). It has been applied for nearly a century as a desensitizing therapy by exposing allergic individuals to gradually increasing doses of allergen extract to induce allergen-specific immune tolerance. The major advantage of allergen immunotherapy over the other medication strategy is that it can provide a long-term relief of symptoms and prevent the progression of disease from mild to severe symptoms (Rice et al., 2018; Dorofeeva et al., 2020). Food immunotherapy has been shown to be effective in the treatment of food allergy, either *via* oral (OIT), sublingual (SLIT), or epicutaneous (EPIT) route (Gernez and Nowak-Węgrzyn, 2017). Although there are various food allergens, most of the clinical trials carried out for food allergen-specific immunotherapy studies are on egg, peanut, and milk allergy (Burks et al., 2018). Peanut-allergic children aged 9 to 36 months received early OIT were able to eat peanut-containing foods at 19-fold higher rates acceptable safety profile than matched standard-care controls (Vickery et al., 2017). Another clinical trial carried out at two US centers with a longer treatment period (5 years) demonstrated that OIT modified the peanut-specific immune response in all subjects that finished the study (Vickery et al., 2014). In an egg OIT study, egg OIT was proven to be effective for desensitization in almost all subjects, although only 31% remained tolerant after 3-month of egg avoidance (Caminiti et al., 2015). In terms of milk OIT clinical trial, 30 children aged 24–36 months with cow’s milk allergy treated with milk OIT. Results showed that 90% of OIT-treated children had become completely tolerant *vs.* 23% of placebo-treated children (Martorell et al., 2011). In addition to single food immunotherapy, multi-food OIT also catches clinicians’ attention. Nadeau et al. investigated the safety of multi-food OIT. In this study, 25 patients were allergic to multiple foods and 15 patients were only allergic to peanut. After these two groups of patients treated with multi-food OIT or peanut OIT respectively, the data revealed that rates of adverse reactions were similar between multi-food OIT or peanut OIT treatments (Bégin et al., 2014). However, these studies involved small sample sizes. Larger, randomized studies are required to continue to test safety and efficacy of food immunotherapy.

Despite of its proven effect, immunotherapy has its own limitations, which, to some extent, impedes its wide application in the clinic. The long duration of treatment with multiple injections results in poor compliance of allergic patients. Besides there is also a risk of side effects, for example, pruritus and swelling at the injection site (Fujita et al., 2012; Rodriguez-Dominguez et al., 2020). A third obstacle in allergen immunotherapy lies in that it is difficult to obtain high quality allergen extracts for the formulation of vaccines (Rodriguez-Dominguez et al., 2020).

## Future Perspective

Along with continuing seeking allergens, targeting at the microbiome community is a promising tactic for the management of allergic diseases.

### Gut Microbiota-Targeted Therapies

Manipulation of intestinal microbial composition through the external intervention has initiated a new era in medical sciences, which could be a novel therapeutic approach for allergic diseases. To achieve this, a variety of strategies could be applied.

Dietary recommendation is a popular intervention to modulate the gut microbiota, however to which degree the microbiota is affected by is still unclear yet and it is not easy for people to keep a restricted diet for a long time (Turnbaugh, 2020). Instead, future study could focus on how gut microbiota regulate allergic reactions and uncover specific bacteria or enzymes that could be targeted at.

Probiotics or prebiotics intervention also draws great attention of researchers. Prebiotics are defined as “a non-digestible food ingredient that beneficially affects the host by selectively stimulating the growth and/or activity of one or a limited number of bacteria in the colon” (Roberfroid, 2000). They mainly act as food for human microflora. Several studies have suggested a potent immunomodulatory activity of certain bacterial strains (Kang and Im, 2015). However, the available results from different studies are contradictory in terms of the effectiveness of probiotics or prebiotics in reducing the risk of allergic disorders (Nadeem et al., 2019). Therefore, the use of probiotics or prebiotics to manipulate the intestinal bacteria for the management of allergic diseases still needs further investigation with rational experimental design.

Engineered bacteria against allergic reaction could be another advanced tactic, which has already been applied in a number of illnesses, such as inflammatory bowel diseases and autoimmune disorders (Piñero-Lambea et al., 2015). Engineered microbe has an array of latent functions, from producing therapeutic molecules to modulating immune responses which demonstrated a great potential in the clinic (Ainsworth, 2020).

Hopefully we could design microbiome-based therapies as suggested by Peter J. Turnbaugh and pave the way for new medication principles (Turnbaugh, 2020). We hope, by restoring the disturbed microbiome functionality, to hinder the growth of allergic diseases with the joint efforts of clinicians, biologists, and statisticians.

### Integration of Rich Data

With the power of various high-throughput techniques, we are able to study microbiota from diverse angles, which would provide us with a complete insight of the role of intestinal microbiome on pathophysiological processes of hosts (Cheng et al., 2019). Integration of multi-omics techniques and to mine the data obtained to get relevant information thoroughly would be a very exciting direction in the field of gut microbiota and human health. With non-targeted strategy, we could explore the compositional variations across the entire mammalian microbiome without prior hypothesis, which facilitated us to seek out the change patterns of total microbiome as well as the variations of host responses (Zhao, 2013). Besides, with this approach, we could also discover the microbial species, proteins, or metabolites that are associated with allergic disorders. This area would be very challenging and meaningful. However, this area would not be successful without proper bioinformatics tools to explore these large multi-omics data which include both supervised and unsupervised statistical approaches. We expect that some key findings or new aspects concerning the complex role of intestinal microbes in allergic disease can be made with this approach.

### Determination of Causative Relation

With current available evidence from both human and animal studies, we could confidently state that there is an association between gut microbiota alteration and allergic disease. However, there are still questions remained to be answered if we would like to apply gut microbiome modulation in the clinic. The most important one is to clarify the causation relationship between specific bacterial strains and allergic disease pathology, which is also the fundamental requirement. In other words, only when we are able to define the causative relation between intestinal microbiome and allergic diseases, can we come up with efficient microbe-based medication remedies to control allergic diseases. Studies must be designed following the four criteria in Koch’s postulates, which has been set to evaluate whether a microbe causes the disease (Segre, 2013). Longitudinal study could be a good choice which requires repeated measurement of particular individual over a certain period of time (Caruana et al., 2015). This kind of study allows us to monitor the disease progression, providing us with the characteristic shifting information of gut microbes over time alongside disease development for the target population as a whole or individual. More intriguingly, gnotobiotic animals, which only contain known strains of bacteria, represents an extraordinary opportunity to uncover the causative relation between specific bacterial strains and allergic diseases (Martín et al., 2016).

## Conclusion

Microorganisms living inside the gut have been no doubt implicated in allergic diseases which have etiologies (Tilg and Moschen, 2014). Our understanding of the relationship between allergic diseases and gut microbiota has been advanced greatly not only by microbiome analysis, but also by data generated by other “-omics” techniques. Acquiring a clear and deep knowledge of the role of gut microbes in the pathology of allergic diseases will surely pave the way for a more rational means to deal with allergic diseases. As mentioned above, this area will be driven by the progress in analytical techniques, study design, and data interpretation strategies. Ultimately, we hope, by deciphering the role of gut microbiome, new medication remedies for the management of allergic diseases will be discovered.

## Author Contributions

YW and J-LS conceived the task. PH, J-QG, L-SL, YW, and J-LS performed the review and collected original studies. PH, J-QG, and L-SL wrote the first draft of the manuscript. YW, J-LS, X-YW, and H-TW revised the manuscript. CC contributed to language editing and final revision. All authors contributed to the article and approved the submitted version.

## Funding

The work was supported by the National Natural Science Foundation of China (Nos. 81971515, and 81973290), CAMS Innovation Fund for Medical Sciences (CIFMS, No. 2016-I2M-3-011 and 2016-I2M-1-003), the Beijing Key Laboratory of Non-Clinical Drug Metabolism and PK/PD study (Z141102004414062, China), the National Megaproject for Innovative Drugs (Nos. 2018ZX09711001-002-002 and 2018ZX09302015, China), Beijing Natural Sciences Fund Key Projects (NO. 7181007), the Fundamental Research Fund for the Central Universities of Peking Union Medical College (No. 3332020037), and Beijing Municipal Administration of Hospitals clinical medicine development of special funding support (ZYLX201826).

## Conflict of Interest

The authors declare that the research was conducted in the absence of any commercial or financial relationships that could be construed as a potential conflict of interest.
